# Ultraviolet B Light Emitting Diodes (LEDs) Are More Efficient and Effective in Producing Vitamin D_3_ in Human Skin Compared to Natural Sunlight

**DOI:** 10.1038/s41598-017-11362-2

**Published:** 2017-09-13

**Authors:** T. A. Kalajian, A. Aldoukhi, A. J. Veronikis, K. Persons, M. F. Holick

**Affiliations:** Boston University School of Medicine, Boston Medical Center, Section Endocrinology, Diabetes, Nutrition and Weight Management, Department of Medicine, Vitamin D, Skin, and Bone Research Laboratory, Boston, MA 02118 USA

## Abstract

Vitamin D, the sunshine vitamin is important for health. Those with fat malabsorption disorders malabsorb vitamin D and thus must rely on cutaneous production of vitamin D_3_. Vitamin D_3_ is generated secondary to exposure to ultraviolet B (UVB) radiation (whether from the sun or from an artificial source). Light emitting diodes (LEDs) have been developed to emit ultraviolet radiation. Little is known about the efficiency of UVB emitting LEDs tuned to different wavelengths for producing vitamin D_3_ in human skin. Ampoules containing 7-dehydrocholesterol were exposed to a LED that emitted a peak wavelength at 293, 295, 298 or 305 nm to determine their efficiency to produce previtamin D_3_. The 293 nm LED was best suited for evaluating its effectiveness for producing vitamin D in human skin due to the shorter exposure time. This LED was found to be 2.4 times more efficient in producing vitamin D_3_ in human skin than the sun in less than 1/60^th^ the time. This has significant health implications for medical device development in the future that can be used for providing vitamin D supplementation to patients with fat malabsorption syndromes as well as patients with other metabolic abnormalities including patients with chronic kidney disease.

## Introduction

The beneficial role of ultraviolet radiation on bone health began in 1919 when Huldschinsky reported that children who were exposed to a mercury arc lamp showed significant radiologic improvement of their rickets several months later. He noted that exposure to one forearm of a child with rickets displayed the same radiologic improvements in the other arm not exposed to the mercury arc lamp^1^. He concluded that something was produced in the skin that entered the circulation causing widespread improvements in bone mineralization in children with rickets. The connection with sunlight was made when Hess *et al*.^2^ exposed rachitic children to the sun in New York City from half an hour to several hours and reported significant improvements in their rickets^2^.

It is now recognized that during exposure to sunlight, ultraviolet B (UVB) radiation between 290–315 nm penetrates into the skin and is absorbed by 7-dehydrocholesterol (7-DHC)^3–5^. This results in the cleavage of the bond between C9 and C10 to form *cis-cis* previtamin D_3_. The action spectrum for the production of previtamin D_3_ revealed that the most efficient wavelengths were 298 ± 2 nm^4^. Once formed, previtamin D_3_ is thermodynamically unstable, and the triene system rearranges into a more thermodynamically stable form, vitamin D_3_^1, 6–10^. The phospholipid bilayer of the keratinocyte cell membrane in human skin plays a major role in this process by maintaining the previtamin D_3_ in a thermodynamically unstable conformer that causes it to rapidly isomerize to vitamin D_3_^7^.

Vitamin D_3_ production in human skin depends on several factors. The size of the area exposed to UV radiation is directly proportional to the amount of vitamin D_3_ produced; the larger the area exposed, the more vitamin D_3_ is produced. For example, sunbathing in a swimsuit can produce an amount of vitamin D_3_ similar to ingesting ~ 20,000 IU of vitamin D^1, 11^. Aging, skin pigmentation, sunscreen use, time of day, latitude, season, and altitude are other factors that affect this vital cutaneous process^1, 6, 8, 10, 11^.

Since the first observations demonstrating that exposure to ultraviolet radiation was effective in curing rickets, various devices emitting ultraviolet radiation have been developed and used to treat and prevent vitamin D_3_ deficiency. The Sperti lamp was available in pharmacies in the United States for the treatment and prevention of rickets in the 1940s^1^. It was a high intensity mercury arc lamp similar to what Huldschinsky had used. A modern version that contains fluorescent tubes that emit ultraviolet radiation was effective in raising blood levels of 25-hydroxyvitamin D [25(OH)D] in healthy adults and in patients with a fat malabsorption syndrome associated with cystic fibrosis^12, 13^. Tanning beds that emit UVB radiation produce vitamin D_3_^14^. Exposure to tanning bed radiation raised blood levels of 25(OH)D in a patient with Crohn’s disease who was unable to absorbed dietary or supplemental vitamin D_3_^15^. Patients with fat malabsorption syndromes including those with inflammatory bowel disease, cystic fibrosis and gastric bypass surgery are at high risk for vitamin D_3_ deficiency and are in need of a user friendly device that can promote the cutaneous production of vitamin D_3_^8, 16^.

UV wavelengths between 290–300 nm were found to be the most efficient for vitamin D_3_ production in human skin and human skin equivalent models^4, 5^. With the advancement of gallium nitride LED (Light Emitting Diode) technology that emits UV radiation, it is now possible to manufacture LEDs that are efficient and suitable for a wide range of commercial uses including for sterilization^17, 18^. These LEDs can be tuned to emit the desired wavelengths including those that can theoretically can convert 7-DHC to previtamin D_3_ in human skin.

Barnkob *et al*. investigated the efficiency of LEDs with different peak wavelengths in producing vitamin D_3_ in pig skin^19^. They found that vitamin D_3_ could be produced when pig skin was irradiated with LEDs that had peak wavelengths between 292–300 nm; 296 nm was found to be the most effective for vitamin D_3_ production. Morita *et al*. exposed mice to LEDs with peak wavelengths between 268 and 316 nm two times per week for 4 weeks (8 doses in total). They reported that serum levels of 25(OH)D significantly increased in exposed mice compared to controls regardless of the LED’s wavelength^20^.

Data regarding the efficiency of UVB emitting LEDs for producing vitamin D_3_ in human skin is lacking. This study had two objectives. First, to determine the efficiency of previtamin D_3_ production in ampoules containing 7-DHC following exposure to LEDs emitting different wavelengths to determine which one was most effective and efficient. The second objective was to compare the efficiency of vitamin D_3_ production in human skin exposed to the most effective and efficient LED to sunlight.

## Methods

### Equipment

**Figure 1:**
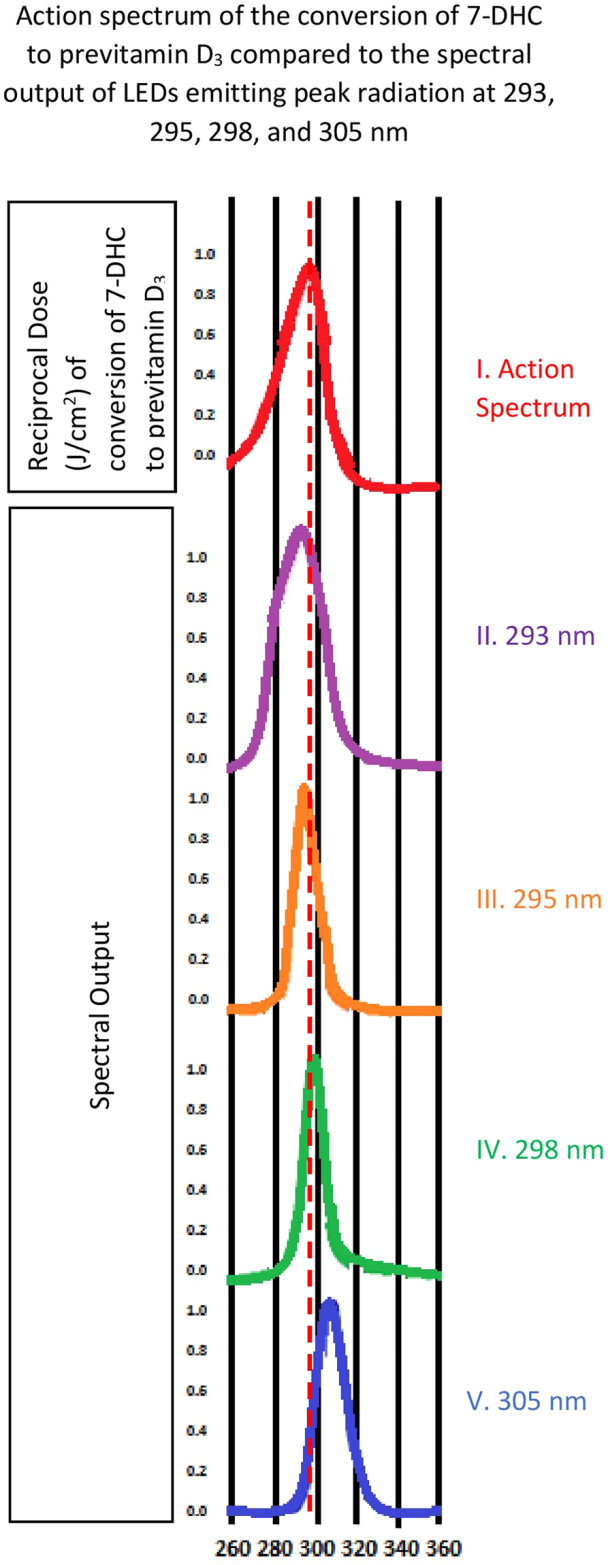
The action spectrum for the production of previtamin D_3_ in human skin (**A**) and the spectral output of the various LEDs that were evaluated (**B**–**E**). The dashed line represents the peak wavelength of 297.5 nm.

Four LEDs with different peak wavelengths, 293, 295, 298, and 305 nm, were used in this study (Fig. 1).The 293 nm LED was obtained from RayVio (Hayward, CA, USA), 295 nm and 305 nm was from Sensor Electronic Technology, Inc (SETi, Columbia, SC, USA), and 298 nm was from DOWA Electronic Materials Company, Ltd (Chiyoda-Ku, Tokyo, Japan). The LEDs were powered with a power supply (supplied by RayVio) set to 50 V and 10 mA for all the experiments. The energy output of each LED was measured using a UVB meter, Solarmeter (Solar Light Company, Inc, Glenside, PA). This meter estimates the output in terms of Minimal Erythemal Dose (MED) per hour which is equivalent to 15.6 mJ/cm^2^/hr.

The ampoules used in this experiment were borosilicate ampoules from Wheaton Inc. (Millville, NJ USA) containing 50 µg/mL of 7-DHC dissolved in 1 mL of ethanol as previously described^12^. The skin samples used were surgically obtained from the Plastic Surgery Department at Boston Medical Center. Each skin sample was cut to have a surface area of approximately 4 cm^2^. This study was categorized as an approved exempt study by Boston University Medical Campus Institutional Review Board (BUMC IRB). All methods in this study were carried out in accordance with the guidelines and regulations issued by our IRB. Due to the exempt nature of this study, all of the discarded samples were deidentified and therefore informed consent was not required from those providing skin samples.

### Ampoule and Skin Exposure to Various LEDs and Sunlight

For UV irradiation, the samples (ampoule or skin) were placed in a quartz dish on top of a plastic apparatus with a 1 cm^2^ opening in the center focused 10.0 mm ± 1.0 mm above the top of the LED. The ampoule or ~4 cm^2^ piece of skin was placed over the 1 cm^2^ opening. To ensure radiation of the entire piece of skin, this process was repeated for the unexposed areas so that the entire skin sample was exposed to the same amount of UVB radiation.

To determine which LED was most efficient in converting to previtamin D_3_, the energy output for each LED was measured using the Solarmeter to calculate the exposure time needed for each LED to reach 46.8 mJ/cm^2^ (equivalent to 3 MEDs). Three ampoules were exposed for each respective calculated time to determine the efficiency of each LED in converting 7-DHC to previtamin D_3_. The LED with the highest percent conversion and shortest/least exposure time was selected for additional experiments for a comparative analyses between the LED and sun exposure using human skin samples. Each skin sample that came from one patient was cut in half; one half was exposed to the LED and the other half was exposed to sunlight in Boston in October at noon time for the same energy exposure.

### Sample Analysis

#### a. Ampoules

Immediately after the allotted time of exposure, 200 µl was recovered from each ampoule and transferred to a test tube and dried under nitrogen gas. The dried samples were re-dissolved in 1 mL 0.8% isopropyl alcohol (IPA) in hexane to be analyzed with a straight phase High-Pressure Liquid Chromatography (HPLC) at a flow rate of 1.5 mL/min as previously described^12^.

#### b. Skin

After the skin was irradiated, it was submerged in water at 60 °C for 1 minute^12^. The epidermis was separated from the dermis using a scalpel to scrape the layer off. The dermis was discarded and the epidermis was placed in 2.5 mL of 8% ethyl acetate in hexane and sonicated for 10 seconds. The samples were then incubated overnight at 50 °C to allow the conversion of previtamin D_3_ to vitamin D_3_. This process facilitated the separation of lipid contaminants that migrated near where previtamin D_3_ eluted, permitting the quantitation of the vitamin D_3._ Therefore, the vitamin D_3_ content was considered as the previtamin D_3_ equivalent observed in ampoules. After the overnight incubation the cellular particulates remained at the bottom and the solution was decanted to a test tube and dried under nitrogen gas. The samples were re-suspended in 1 mL of 0.8% IPA in hexane, centrifuged to remove any remaining particulates and the supernatant was dried under nitrogen, and resuspended in 140 µl of 0.8% IPA in hexane and analyzed on a straight phase HPLC at a flow rate of 1.5 mL/min.

### Statistical Analysis

Descriptive statistics were used to present the data, mean and standard deviation. No significant difference in mean previtamin D_3_ percent conversion was expected between the 293, 295, and 298 nm LED, so no formal statistical testing was done to compare them. However, the percent conversion for the 305 nm LED was expected to be significantly different compared to the other LEDs based on the published previtamin D_3_ action spectrum^4^.

## Results

### Wavelength efficiency in previtamin D_3_ production in ampoules

Four ampoules containing 7-DHC were irradiated for a time that was equivalent to 46.8 mJ/cm^2^ (Table 1). The mean percent conversion of 7-DHC to previtamin D_3_ was similar for the 293, 295, and 298 nm LEDs. However, the percent conversion for the 305 nm LED was more than 90% lower compared to other LEDs (*P*-value < 0.001; Table 1). The 293 nm LED was able to generate the same amount of previtamin D_3_ in less than half the time compared to the other LEDs. This was also apparent when evaluating the percent conversion of 7-DHC to previtamin D_3_ for the same period of time of 2.55 minutes for each of the LEDs. For example only 0.47% of 7-DHC would be converted to previtamin D_3_ with the 305 nm LED compared to 11.2% with the 293 nm LED. Therefore the 293 nm LED was selected for the comparison of the vitamin D_3_ production efficiency with natural sunlight.

**Table 1:** Exposure Time and percent conversion of 7-DHC to previtamin D_3_.

LED peak Wavelength (nm)	Exposure Time (minutes)^a^	Mean percent conversion ± SD	Mean percent conversion ± SD @ 2.55 min^c^
293 (Rayvio)	2.55	11.2% ± 0.18%	11.2% ± 0.18%
295 (SETI)	8.62	10.6% ± 0.24%	2.18% ± 0.07%
298 (DOWA)	5.65	11.1% ± 0.25%	5.00% ± 0.11%
305 (SETI)	5.43	1.0% ± 0.24%^b^	0.47% ± 0.11%

^a^Time to reach 46.8 mJ/cm^2^. ^b^Significantly different from other LED (*P*-value < 0.001). ^c^Percent conversions standardized for shortest exposure time to reach 46.8 mJ/cm^2^ (2.55 min).

### Efficiency of Sunlight vs LED on Vitamin D_3_ Production in Human Skin

**Figure 2:**
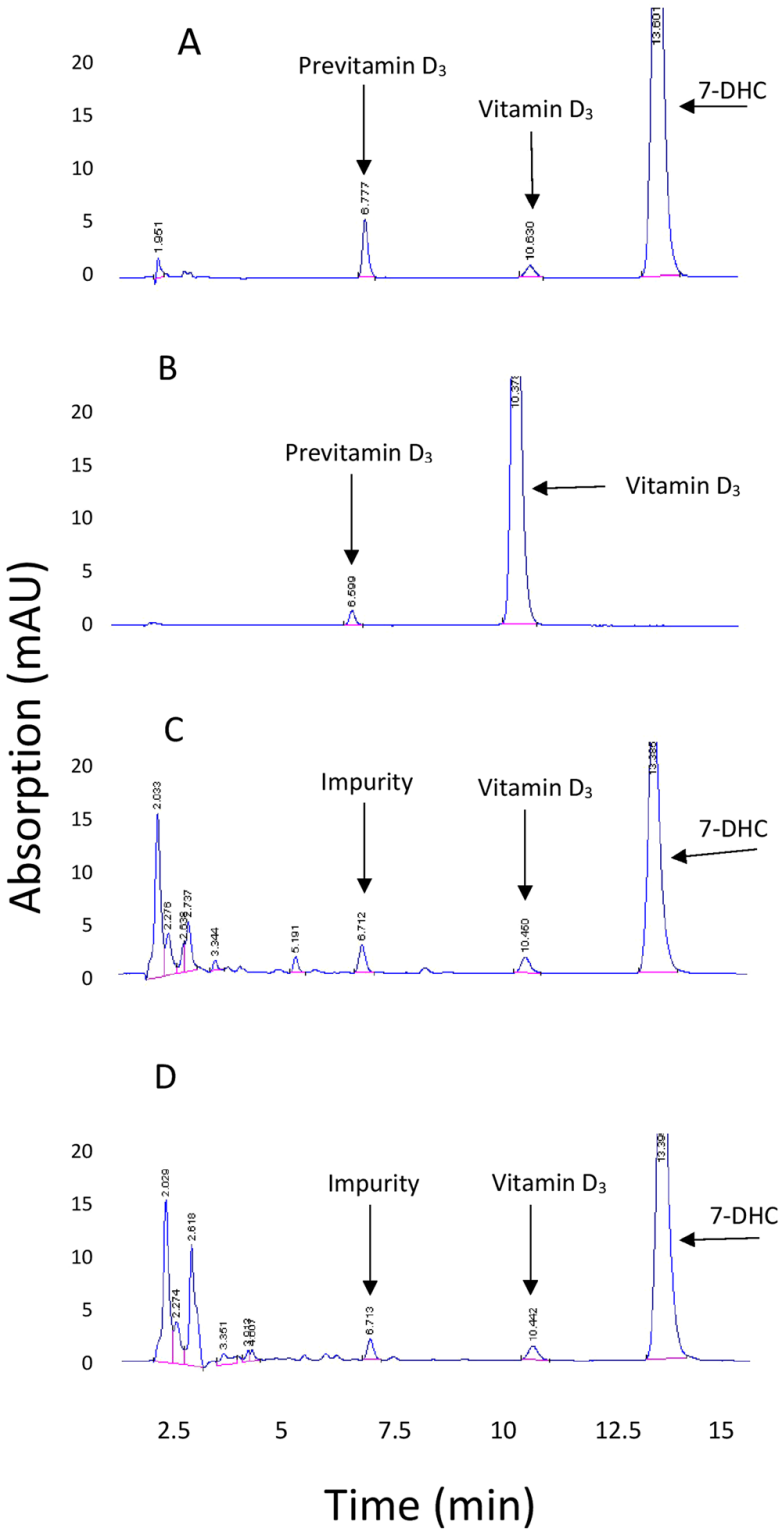
Chromatograms of ampoules containing 7-DHC and exposed to ultraviolet radiation (**A)** and standards of previtamin D_3_ and vitamin D_3_ (**B**). 2 C and 2D are chromatograms of lipid extracts of human skin after exposure to a 293 nm LED (**C**) and sunlight (**D**) followed by incubation overnight.

**Figure 3:**
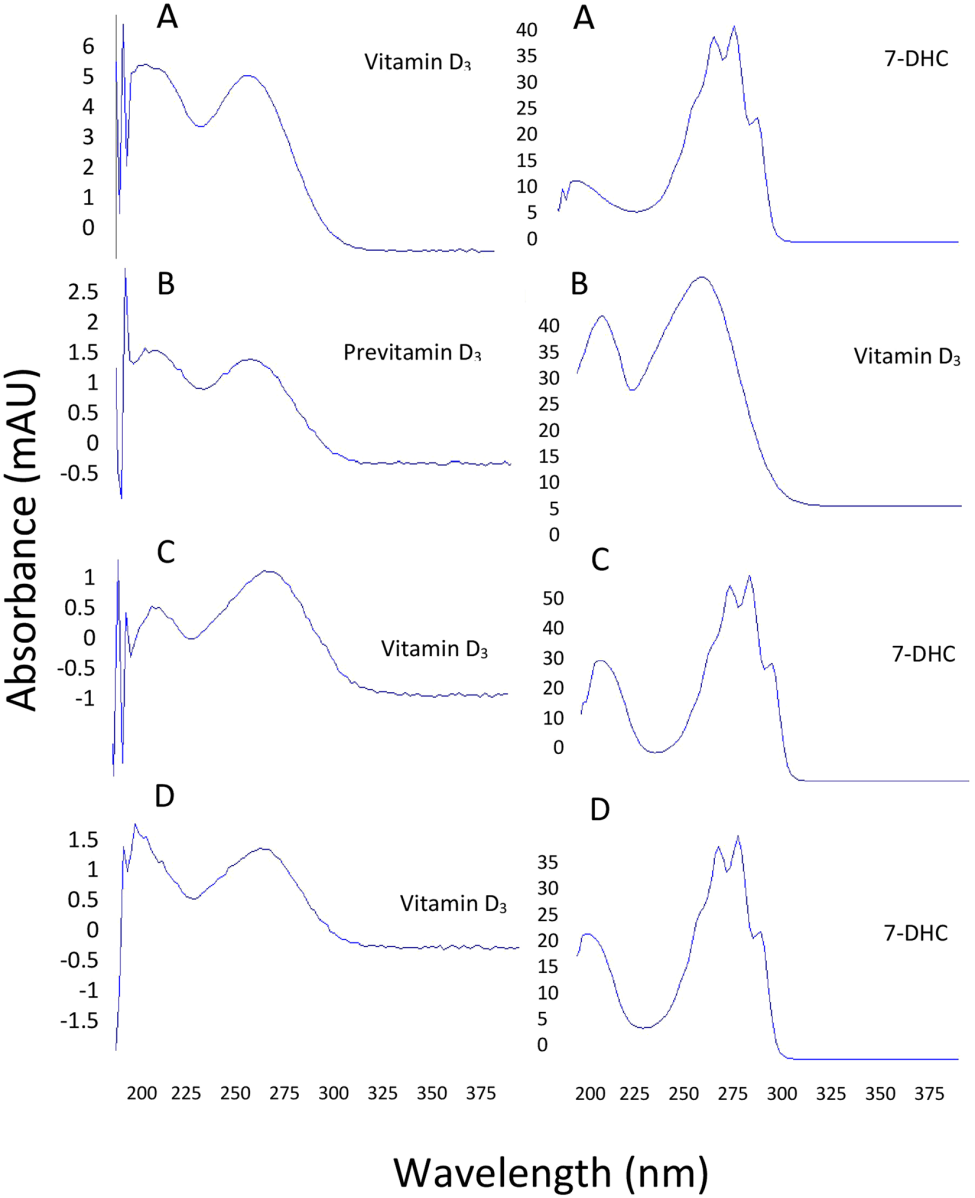
UV absorption spectrums of vitamin D_3_, previtamin D_3_, and 7-DHC obtained from ampoules (**A** and **B**) and from human skin after exposure to a 293 nm LED (**C**) and sunlight (**D**).

Fitzpatrick skin type II samples from different donors were exposed to the sun on different days for 32.15 minutes (11.7 mJ/cm^2^; 0.75 MED) and 1 hour (37.4 mJ/cm^2^) at 12 pm in the middle of October. The other half of the skin sample was exposed to the 293 nm LED on the same day for 1.36 min and 2.73 min to obtain the same energy exposure for the 30 minute (11.7 mJ/cm^2^) and one hour sun exposure (37.4 mJ/cm^2^) respectively. Figure 2 presents representative chromatograms and Fig. 3 presents the UV absorption spectrums that confirmed the identification of the peaks, of an ampoule exposed to UV radiation showing 7-DHC and previtamin D_3_ (A) and the thermal conversion of previtamin D_3_ to vitamin D_3_ (B). Figure 2C and D are representative chromatograms of human skin exposed to the 293 nm LED and sunlight respectively demonstrating the lipid contaminants that migrated near where previtamin D_3_ migrated. The UV absorption spectrum for the contaminant was different than previtamin D_3_.

An evaluation of the efficiency of the 293 nm LED to produce vitamin D_3_ in type II skin compared to sunlight revealed that the LED was greater than 2 fold more effective (Table 2). It took only 0.52 minutes for the 293 nm LED to emit 11.7 mJ/cm^2^ which resulted 1.2% of the 7-DHC to be converted to vitamin D_3_. Exposure to the same amount of energy from the sun took 32.5 minutes and only 0.5% of the 7-DHC was converted to vitamin D_3_. When a separate type II sample was exposed to 37.4 mJ/cm^2^ on a different day similar results were obtained. It took only 2.73 minutes for 1.9% of 7-DHC to be converted to vitamin D_3_ compared to 60 minutes of sun exposure converting 1.3% of 7-DHC to vitamin D_3_. To determine the effect of increased skin pigmentation on the cutaneous production of vitamin D by the LED compared to sunlight, skin type III from 2 subjects was exposed to 31.2 mJ/cm^2^ from the 293 LED and sun on the same day. It took 92.2 minutes of sun exposure to convert 0.6% of 7-DHC to vitamin D_s_ compared to only 2.47 minutes to convert 1.3 and 1.7% of the 7-dehydrocholesterol to vitamin D_3_ in the type III skin samples.

**Table 2:** Percent conversions of 7-DHC to previtamin D_3_ in skin Type II and skin Type III following LED/Sun exposure.

Skin Type	Energy Exposure (mJ/cm^2^)	Percent Conversion of 7-DHC to vitamin D_3_			
		LED	Exposure Time (min)	Sun	Exposure Time (min)
Type II	11.7	1.2%	0.52	0.5%	32.15
Type II	37.4	1.9%	2.73	1.3%	60.00
Type III	31.2	1.7%	2.47	0.6%	92.30
Type III	31.2	1.3%	2.47	0.6%	92.30

## Discussion

We evaluated several LEDs of varying wavelengths to determine which was most desirable to develop for human trials. We found that the 293 nm LED, provided by Rayvio, was best suited for human trials due to a more than 50% shorter exposure time to produce the same amount of vitamin D_3_ as the other LEDs. This LED was also found to be 2.4 and 2.5 times more efficient in producing vitamin D_3_ in Type II and Type III human skin respectively compared to being exposed to the same amount of energy from the sun in a much shorter time. For example the suberythemal exposure of the skin type II to 0.75 MED from the 293 LED produced 2.3 IUs cm^2^ (5.63 ng/cm^2^) in one half minute compared to a less than half the amount (0.9 IUs; 2.35 ng/cm^2^) vitamin D_3_ when the same skin sample was exposed to the sun for more than 60 times longer time (32.15 min). Assuming an average adult body surface area 1.7 m^2^ ^21^, a 0.75 MED exposure of the whole body to the 293 nm LED would generate 39,100 IUs. The Institute of Medicine recommends that for most children and adults their requirement is 600 IUs of vitamin D a day. This would translate into exposing 260 cm^2^ (40.3 in^2^) of the skin surface to the 293 nm LED for approximately one half minute. This is compared to generating 15,300 IUs exposed to 0.75 M ED of sunlight. To generate 600 IUs of vitamin D_3_ would require 666 cm^2^ (103 in^2^) of the skin surface to be exposed to approximately 30 minutes of sunlight.

Vitamin D production in human skin following sun exposure depends on the position of the sun in the sky, or the zenith angle^6, 8, 11, 22^. The amount of UV radiation reaching the earth’s surface increases when the zenith angle decreases. Moreover, the zenith angle depends on the latitude, season, and time of the day^1, 23, 24^. As a result, the optimal times for vitamin D production in human skin is in the summer between the hours of 10 AM-3PM^1, 24, 25^. However because time of day, season, latitude, altitude, and weather conditions can all influence the cutaneous production of vitamin D_3_ it is difficult to obtain an adequate amount of vitamin D_3_ from sun exposure without some guidance. Furthermore it is often unrealistic to be outdoors between 10 AM and 3 PM because of working schedules. Therefore, LEDs would offer an alternative efficient way of providing the user with a defined suberythemal amount of UVB radiation for producing their vitamin D requirement. These LEDs can also be developed for treating and preventing recurrent vitamin D deficiency in patients who are unable to absorb vitamin D through their gastrointestinal tract due to fat malabsorption syndromes^15^. Another use could be in patients with chronic kidney disease. It was observed that patients with end-stage chronic kidney disease who were exposed to UVB radiation were able to improve their vitamin D status as well as increase their blood levels of 1,25-dihydroxyvitamin D_3_ and decrease their parathyroid hormone levels thereby improving their calcium and bone metabolism^26^. In addition, these patients required less erythropoietin to maintain their blood hematocrit and had significant improvement in their cardiovascular status^26^.

However, prolonged exposure to UV radiation from the sun increases the risk for non-melanoma skin cancer^27^. This occurs mainly due to DNA damage from wavelengths that are absorbed by DNA that lead to the formation of DNA damage products including cyclobutane pyrimidine dimers (CPD)^27,28^. Masuma *et al*. studied CPD formation after exposing rat pheochromocytoma cell line, PC 12 cells, to different UV wavelengths (250 nm–310 nm). They found that CPD formation to be highest after 250 nm exposure and lowest after 310 nm exposure^29^. Thus, the longer the wavelength, the lesser the DNA damage. Furthermore, Felton *et al*. reported that suberythemal exposure to simulated sunlight in the UK was effective in raising blood levels of 25-hydroxyvitamin D_3_ (a measure of vitamin D status) while at the same time demonstrating enhanced DNA repair mechanisms that minimized DNA damage from the exposure^27^ suggesting that humans have adapted to sensible sun exposure^30^. Although the 305 nm would have been thought to be the most desired due to its lower energy, because of the marked decrease in quantum efficiency, there was a more than 10 fold decrease in the production of previtamin D_3_ compared to the other LEDs. It would likely require 10 times more 305 nm photons to generate the same amount of previtamin D_3_ as the 293 nm LED. Therefore another advantage of the 293 nm LED may be reduced risk of skin damage when compared to exposure to sunlight or the 305 nm LED.

LEDs can be manufactured with any desired wavelength that has a narrow range. It is possible to have an LED with a wavelength that can maximally produce vitamin D_3_ more efficiently than sunlight. These LEDs can be used for different functions including vitamin D_3_ production for patients with malabsorption syndromes. The LED can also be incorporated into wearable gadget devices to provide individuals with their daily vitamin D_3_ requirement. Moreover, LEDs can be used for vitamin D_3_ for patients with chronic kidney disease (CKD). It has been showed that UVB exposure was superior to oral supplements in elevating serum levels of 25(OH)D in patients with CKD^26^.

## Conclusion

The optimal range of LEDs emitting UVB radiation for vitamin D production was found to be between 293 and 298 nm. The 293 nm LED was found to be 2.4 times more efficient in converting 7-DHC to previtamin D_3_ in human skin than the sun, thus improving its safety profile. This data provides helpful information for medical device development in the future that can be used for vitamin D supplementation in both healthy and diseased individuals.

## References

[1] Wacker M, Holick MF (2013). Sunlight and Vitamin D: A global perspective for health. Dermatoendocrinol..

[2] Hess AF, Ungerm LJ (2016). The cure of infantile rickets by artificial light and by sunlight. Exp Biol Med..

[3] Lehmann B, Genehr T, Knuschke P, Meurer M, Pietzsch J (2001). UVB-induced conversion of 7-dehydrocholesterol to 1α,25-dihydroxyvitamin D_3_ in an *in vitro* human skin equivalent model. J Invest Dermatol..

[4] MacLaughlin JA, Anderson RR, Holick MF (1982). Spectral character of sunlight modulates photosynthesis of previtamin D_3_ and its photoisomers in human skin. Science..

[5] Nemanic MK, Whitney J, Elias PM (1985). *In vitro* synthesis of vitamin D_3_ by cultured human keratinocytes and fibroblasts: action spectrum and effect of AY-9944. Biochim Biophys Acta..

[6] Holick MF (1981). The cutaneous photosynthesis of previtamin D3: a unique photoendocrine system. J Invest Dermatol..

[7] Holick MF, Tian XO, Allen M (1995). Evolutionary importance for the membrane enhancement of the production of vitamin D_3_ in the skin of poikilothermic animals. Proc Natl Acad Sci..

[8] Holick MF (2007). Vitamin D deficiency. N Engl J Med..

[9] Holick MF (2012). Vitamin D: A D-lightful solution for good health. J Med Biochem..

[10] Holick MF, Chen TC (2008). Vitamin D deficiency: a worldwide problem with health consequences. Am J Clin Nutr..

[11] Nimitphong H, Holick MF (2013). Vitamin D status and sun exposure in southeast Asia. Dermatoendocrinol..

[12] Dabai N, Pramyothin P, Holick MF (2012). The effect of ultraviolet radiation from a novel portable fluorescent lamp on serum 25-hydroxyvitamin D_3_ levels in healthy adults with Fitzpatrick skin types II and III. Photodermatol., Photoimmunol., Photomed..

[13] Chandra P (2007). Treatment of vitamin D deficiency with UV light in patients with malabsorption syndromes: a case series. Photodermatol Photoimmunol Photomed..

[14] Tangpricha V (2004). Tanning is associated with optimal vitamin D status (serum 25-hydroxyvitamin D concentration) and higher bone mineral density. Am J Clin Nutr..

[15] Koutkia P, Lu Z, Chen TC, Holick MF (2001). Treatment of vitamin D deficiency due to Crohn’s disease with tanning bed ultraviolet B radiation. Gastroenterology..

[16] Margulies SL, Kurian D, Elliott MS, Han Z (2015). Vitamin D deficiency in patients with intestinal malabsorption syndromes – think in and outside the gut. J Dig Dis..

[17] Kneissl, M. *et al*. Advances in group III-nitride-based deep UV light-emitting diode technology. *Semiconductor Science and Technology*. **26** (2011).

[18] Nakamura S, Krames MR (2013). History of Gallium-Nitride-Based Light-Emitting Diodes for Illumination. Proceedings of the Ieee..

[19] Barnkob, L. L., Argyrak, I. A., Petersen, P. M. & Jakobsen, J. Investigation of the effect of UV-LED exposure conditions on the production of vitamin D in pig skin. *Food Chem.* **212**, 386–91 (2016). 10.1016/j.foodchem.2016.05.155 27374546

[20] Morita D (2016). Short-range ultraviolet irradiation with LED device effectively increases serum levels of 25(OH)D. J Photochem Photobiol B..

[21] Sacco JJ, Botten J, Macbeth F, Bagust A, Clark P (2010). The average body surface area of adult cancer patients in the UK: a multicentre retrospective study. PLOS ONE..

[22] Webb, A. R., Kline, L. & Holick, M. F. Influence of season and latitude on the cutaneous synthesis of vitamin D: Exposure to winter sunlight in Boston and Edmonton will not promote vitamin D synthesis in human skin. *J Clin Endo Metab*. **67**(2), 373–378 (1988). 10.1210/jcem-67-2-373 2839537

[23] Chen, T. C. *et al*. Factors that influence the cutaneous synthesis and dietary sources of vitamin D. *Arch Biochem Biophys*. **460**, 213–7 (2007). 10.1016/j.abb.2006.12.017 PMC2698590 17254541

[24] Holick, M. F. Biological effects of sunlight, ultraviolet radiation, visible light, infrared radiation and vitamin D for health. *Anticancer Res.* **36**, 1345–1356 (2016). 26977036

[25] Webb, A. R. Who, what, where and when-influences on cutaneous vitamin D synthesis. *Prog Biophys Mol Biol*. **92**, 17–25 (2006). 10.1016/j.pbiomolbio.2006.02.004 16766240

[26] Krause, R., Roth, H. J., Kaase, H., Stange, R. & Holick, M. F. Vitamin D status in chronic kidney disease - UVB irradiation is superior to oral supplementation. *Anticancer Res.* **36**, 1397–401 (2016). 26977042

[27] Felton, S. J. *et al*. Concurrect beneficial (vitamin D production) and hazardous (cutaneous DNA damage) impact of low-level summer sunlight exposures. *Br J Dermatol.* **175**(6), 1320–1328 (2016). 10.1111/bjd.14863 PMC5215649 27411377

[28] Pfeifer, G. P. & Besaratinia, A. UV wavelength-dependent DNA damage and human non-melanoma and melanoma skin cancer. *Photochem Photobiol Sci.* **11**, 90–7 (2012). 10.1039/c1pp05144j PMC3289542 21804977

[29] Masuma, R., Kashima, S., Kurasaki, M. & Okuno, T. Effects of UV wavelength on cell damages caused by UV irradiation in PC12 cells. *J Photochem Photobiol B*. **125**, 202–8, doi:10.1016/j.jphotobiol.2013.06.003 (2013). 10.1016/j.jphotobiol.2013.06.003 23856615

[30] Holick. M. F. Can you have your cake and eat it too? The sunlight D-lema. *Br J Dermatol.* **175**(6), 1129–1131 (2016). 10.1111/bjd.15127 27996132

